# The Principal Offices of the Brain and Other Centres

**Published:** 1844-10

**Authors:** 


ART. VIII.—*The Principal Offices of the Brain and other Centres.* By JOSEPH SWAN.—*London,* 1844. 8vo, pp. 32.

THIS pamphlet is obviously intended by its author, as a comprehensive sketch of the laws of nervous agency; and coming from one who has made the anatomy of the nervous system an object of so much industrious and extended study, it has a strong claim upon our attention. It contains an excellent summary of the present state of our knowledge in regard to the functions of this system; and is valuable as proceeding from one so well acquainted with its structure. But we fear that the *form* in which the ideas are expressed, and the mode in which terms are employed, will prevent this summary from being as useful as it might otherwise have been. The language is generally of so peculiarly *abstract* a character, that we have been compelled to use no little exercise of thought, to discover the author's meaning on several occasions. And we must strongly object to the employment of the term perception to "an impression made on the nervous system, of which the animal is not conscious." Perception is the act of *perceiving;* and a being can scarcely be said to perceive that of which it is not conscious. Besides, what is the use of using any other term than impression for the act in question? The consciousness of the impression becomes sensation; and the excitement, by the sensation of that elementary idea of the nature of the object, which is altogether different from the sensation itself, constitutes what is generally and very appropriately termed perception, in this country at least. Moreover, Mr. Swan converts the term sensory, which has hitherto had only an adjective sense in our language, into a substantive; using it, we presume, as the representative of the Latin *sensorium.* This last term, however, is so constantly employed by writers upon physiology aud metaphysics, that we cannot perceive any objection to it; and the introduction of the word sensory in any other mode than that in which it has hitherto (we believe) been universally used, is liable to produce an injurious confusion. We believe, however, that these blemishes, when properly regarded, will not be found to detract seriously from the value of Mr. Swan's pamphlet; and that it may be profitably studied by those, who desire to become acquainted with the general views at present attained on the physiology of the nervous centres. On no other point of importance do we feel called upon to express dissent from him.

